# The Pharmacokinetics of Beta-Lactam Antibiotics Using Scavenged Samples in Pediatric Intensive Care Patients: The EXPAT Kids Study Protocol

**DOI:** 10.3389/fphar.2021.750080

**Published:** 2021-12-10

**Authors:** Stef Schouwenburg, Enno D. Wildschut, M. de Hoog, Birgit C.P. Koch, Alan Abdulla

**Affiliations:** ^1^ Department of Hospital Pharmacy, Erasmus MC, University Medical Center Rotterdam, Rotterdam, Netherlands; ^2^ Department of Pediatric Intensive Care, Erasmus MC – Sophia Children's Hospital, University Medical Center Rotterdam, Rotterdam, Netherlands

**Keywords:** beta-lactam, antibiotics, criticial illness, children, pharmacokinetics, pharmacodynamics

## Abstract

**Background:** Emerging evidence supports the importance of optimized antibiotic exposure in pediatric intensive care unit (PICU) patients. Traditional antibiotic dosing is not designed for PICU patients, as the extreme pharmacokinetic (PK) behavior of drugs threatens the achievement of optimal antibiotic treatment outcomes. Scavenged sampling is a sampling strategy which may have positive implications for routine TDM and PK research, as well as monitoring other biomarkers. EXPAT Kids study was designed to analyze whether current empiric dosing regimens of frequently used beta-lactam antibiotics achieve defined therapeutic target concentrations in PICU patients.

**Methods:** A mono-centre, exploratory pharmacokinetic and pharmacodynamic study was designed to assess target attainment of beta-lactam antibiotics. One hundred forty patients will be included within 24 months after start of inclusion. At various time points serum concentration of the study antibiotic (cefotaxime, ceftazidime, ceftriaxone, cefuroxime, flucloxacillin, and meropenem) are determined. In parallel with these sampling moments, residual material is collected to validate the use of blood of scavenged heparinized astrup syringes for the quantification of antibiotic exposure. The primary outcome is the time that the free (unbound) concentration of the study antibiotic remains above one to four the minimal inhibitory concentration during a dosing interval (100%ƒT > MIC and 100%ƒT>4xMIC). Other included outcomes are disease severity, safety, length of stay, and inflammatory biomarkers.

**Discussion:** Potentially, scavenged sampling may enrich the EXPAT Kids dataset, and reduce additional blood sampling and workload for clinical personnel. The findings from the EXPAT Kids study will lead to new insights in the PK parameters of beta-lactams and consecutive effects on target attainment and clinical outcomes. Is there a need for more precision in dosing? *Netherlands Trial Register Number:* Trial NL9326.

## Introduction

Infectious disease are among the most prevalent causes of mortality in the pediatric intensive care unit (PICU), with a mortality rate up to 50% depending on the site of infection (Dorofaeff et al., 2012). Early initiation of antibiotic therapy has been demonstrated to be the best intervention for severe infections in this population of critically ill patients (Weiss et al., 2015). Additionally, inappropriate dosing of antibiotics has been associated with increased morbidity and mortality in children, and longer PICU stay (Muszynski et al., 2011; Rosa and Goldani, 2014; Zhang et al., 2015).

Studies have demonstrated that current pediatric dosing strategies for beta-lactams fail to achieve pharmacodynamic endpoints, as approximately 95% of pediatric patients achieve subtherapeutic beta-lactam concentrations (Cies et al., 2018; Hartman et al., 2019a). If indeed exposure is suboptimal, titration of appropriate antibiotic dosing might result in increased treatment efficacy, less development of antibiotic resistance, and less drug-induced toxicity (Abdul-Aziz et al., 2015). To determine whether action is required to adjust existing prescribing practices for beta-lactam antibiotics in critically ill pediatric patients, assessment of the current situation at the PICU is warranted.

Standard of care antibiotic dosing regimens are not designed to treat critically ill children due to the complexity of their physiological state. PICU patients are characterised by altered pharmacokinetic parameters, changes in renal function, and are often infected by less susceptible micro-organisms. Observed extreme pharmacokinetic (PK) behavior of drugs poses a significant threat to achievement of optimal clinical outcomes (Roberts et al., 2010). A larger distribution volume and higher clearance in critically ill children might demand an increased dosage or prolonged infusion (Hartman et al., 2020).

Dosing strategies for beta-lactam antibiotics are based upon the minimum inhibitory concentration (MIC) of micro-organisms. Due to unreliability of techniques to determine the MIC and the fact that often no positive culture is available, the epidemiological cut-off value (ECOFF) for a given species and antibiotic are used (Mouton et al., 2018). The MIC_ECOFF_ describes the highest MIC for organisms devoid of phenotypically-detectable acquired resistance mechanisms and defines the upper end of wild-type distribution (EUCAST, 2018).

For data collection, both scheduled and scavenged sampling will be obtained. Scavenging involves the use of residual material of all biological fluids (e.g. blood, liquor, urine, or saliva) which are left over from standard clinical practice (Cohen-Wolkowiez et al., 2012). Importantly, scavenged sampling does not carry any extra burden or risks for the patient.

Timely initiation and target attainment of antimicrobial treatment in infectious disease such as sepsis is warranted, since acute phase pathophysiological changes alter drug PK (Thakkar et al., 2017; Weiss et al., 2020). In this study we want to analyse whether empirical antibiotic dosing regimens of frequently used beta-lactam antibiotics achieve defined pharmacodynamic target (PDT) concentrations in critically ill pediatric patients. Secondly, the association of target attainment with patient characteristics and clinical outcomes will be examined. Lastly, during this study we aim to validate the use of blood of scavenged heparinized astrup syringes for the quantification of antibiotic exposure.

## Methods

The EXPAT Kids protocol is a prospective, mono-centre, observational study which aims to identify whether critical ill patients treated with beta-lactam antibiotics reach target attainment. The study will include 145 patients over a period of 24 months, recruited at the Sophia Children’s Hospital, Erasmus Medical Center, Rotterdam, the Netherlands. See Table 1 for an overview of the EXPAT Kids study procedures.

**TABLE 1:** Study procedures timeline. Time path of enrolment, sampling, and assessments in the study.

	Enrolment	Allocation	Post-allocation	Follow-up
Timepoint	t = 0	t = 0–36 h	t = day 1 till day 5	t = day 7
Start target antibiotic	X	—	—	—
Informed consent	—	X	—	—
Eligibility screen	—	X	—	—
Allocation	—	X	—	—
Collection				
Blood sampling	—	A: trough sample	C: sample during routine morning lab	—
		B: peak sample		
Assessments				
Demographics	X	—		—
Lab data	X	—	X	—
Clinical data, admission data	X	—	X	—
Survival	—	—	—	X

Study antibiotics include cefotaxime, ceftazidime, ceftriaxone, cefuroxime, flucloxacillin, and meropenem.

### Selection of Subjects

All patients admitted to the PICU and given standard of care intravenous (IV) therapy of the study antibiotics will be screened for inclusion. Eligible patients will be identified on a daily basis. Deferred consent is obtained by research staff at a maximum of 24 h after start of study procedures. Since most participants will be minors, their legal representatives will be inquired. If possible, informed consent from the patient is obtained at day five in case of deferred consent by a legal representative.

### In- and Exclusion Criteria

Patients will need to receive IV antibiotic therapy of the study antibiotics which should be aimed for at least 2 days at the time of inclusion. All participants are required to have suitable intra-arterial access to facilitate sample collection, in place through standard of care procedures. Patients will only be included if sampling within 36 h after starts of antibiotic therapy is possible. Patients will be excluded in case of prematurity (<37 weeks old), history of anaphylaxis for study antibiotic, study antibiotic cessation before start of sample collection, and prophylactic use of the study antibiotic.

### Sample Size Calculation

Sample size calculation for the primary objective is based on ƒT > MIC_ECOFF_ PDT attainment prevalence of 60% (95% CI 52–68%), as has been found in the previous EXPAT study on the adult ICU (Abdulla et al., 2020a). For a sample size of 145 patients, an estimated 87 participants are expected to achieve PDT. This amount will be sufficient for the analysis of the primary objective (ƒT > MIC_ECOFF_ and ƒT > 4x MIC_ECOFF_), for which all antibiotics will be pooled.

### Pharmacokinetic Sampling

All collected plasma concentrations of the study antibiotics from which the exact time of blood sampling after administration is known will be used to describe PK profiles. For each patient a trough (t = 0, shortly before dosage) and peak (t = 10–30 min after dosage) blood sample will be drawn during a single IV antibiotic dosage. Additionally, during routine morning (approx. 8:00 h) lab sampling an extra blood sample will be drawn for five consecutive days after obtaining trough and peak samples.

Alongside the above described process, scavenged samples will be obtained through collection of residual material from clinical chemistry and blood gas material from heparinized astrup syringes. These samples will vary in time after dosage.

Total and unbound drug concentrations will be measured in serum by means of a validated LC-MS/MS method in the Erasmus University Medical Center (Erasmus MC) (Abdulla et al., 2017). Scavenged samples from blood gas material will be compared with concurrent scheduled samples using Bland-Altman Analysis.

### Data Collection

All data collected through study procedures will be stored into an eCRF. Laboratory data will include: albumin, conjugated bilirubin, creatinine, C-reactive protein, interleukin-6, procalcitonin, serum liver enzymes, urea, and white blood cell count. Clinical data involve the Pediatric Logistic Organ Dysfunction (PELOD) score, fluid balance, and presence/absence of surgery in the previous 24 h. Additionally, we will collect admission and discharge dates, admission diagnosis, and outcome following discharge/transfer from PICU. The most prevalent and most severe side effects will also be collected. An overview of variables measured in the EXPAT Kids study is presented in Table 2.

**TABLE 2:** List of variables captured in the EXPAT Kids study.

Demographic data
** **Age (in children <1 year also gestational age, postconceptional age)
** **Gender
** **Height
** **Weight
** **PICU ward
Clinical data
** **PICU stay (days) OR date of hospital/PICU admission and discharge
** **Admission diagnosis
** **Comorbidities
** **Sickness severity scores (PELOD)
** **Vasopressors
** **Body temperature variation
** **Blood pressure
** **Heart rate variability – stress/inflammation predictor
** **Presence/absence of surgery within previous 24 h
** **Outcome following discharge/transfer from PICU (alive or deceased)
Organ function and clinical chemistry data
** **Renal function – serum creatinine concentration
** **Liver function – AST, ALT, conjugated bilirubin
** **Fluid balance for total length of stay and previous 24 h
** **Albumin
** **Urea
Antibiotic dosing data
** **Antibiotic (also concomitant antibiotics)
** **Start and end date of the antibiotic treatment
** **Dose and frequency antibiotic therapy
** **Time of dosing and sampling
** **Days of antibiotic therapy
Infection data
** **White blood cell count
** **Interleukin-6
** **Procalcitonin
** **C-reactive protein
** **Known or presumed pathogen (positive blood culture and organisms isolated)

## Data Analysis

### Baseline Characteristics

Demographic and clinical characteristics will be described using standard statistical analysis methods. Descriptive data will be presented as percentages, means ± SD for normally distributed variables, and medians ± interquartile ranges for non-normally distributed variables. To examine differences between groups in categorical variables, Fisher’s exact test will be used. For normally distributed continuous variables, the two-sample Student’s t-test will be used. Otherwise the two-sample Mann-Whitney test will be used. Statistical analyses will be conform previously performed research by our research group (Abdulla et al., 2020a).

### Primary Outcome

The main objective is to determine the prevalence of target attainment for six beta-lactam antibiotics in the early phase after start of therapy in PICU patients. Target attainment for beta-lactam antibiotics is set at 100% of time (T) of the dosing interval in which the unbound (free, ƒ) serum antibiotic concentration remains above the epidemiological cut-off (ƒT > MIC_ECOFF_ and ƒT > 4x MIC_ECOFF_). Both ƒT > MIC_ECOFF_ and ƒT > 4x MIC_ECOFF_ have been described in literature before as key attainment parameters (Roberts et al., 2014).

### Secondary Outcomes

Multiple secondary outcomes were identified, namely: 1) length of PICU stay; 2) concomitant use of other antibiotics; 3) inflammatory biomarkers; 4) serum albumin; and 5) estimated glomerular filtration rate.

We will estimate multivariate binary logistic regression analyses and present the odds ratios (ORs) and 95% confidence intervals (95% CI) for each individual antibiotic. We will include PELOD score at inclusion in the multivariate analysis to control for all our regressions for clinically and relevant baseline characteristics. Multivariate negative binomial regression models examining the association of PDT attainment characteristics with PICU length of stay will be included. For these regressions, we will present the ORs and 95% CI. Statistical significance will be accepted at *p* ≤ 0.05.

In addition to our secondary outcomes, we will develop population PK (popPK) models for the study antibiotics using non-linear mixed effect modelling (NONMEM). Potential bias or over- and underestimation of antibiotic exposure introduced by scavenged sampling will be taken into account and investigated.

### Data Monitoring

Because of the nature of the trial with a low risk of intermediate complications, an independent monitor will visit once during the study period. A percentage of cases will be randomly selected for verification by the independent monitor. Informed consent, source data, and reported serious adverse events (SAEs) are reviewed for errors. The data will be pseudonymised when stored in the database and then used for analysis.

### Ethics and Dissemination

This trial was approved by the Medical Ethics Committee of the Erasmus Medical Centre in Rotterdam, the Netherlands (registration number MEC-2021-0173). For every significant change to the protocol, an amendment will have to be approved by the local medical ethics committee. Written deferred consent will be obtained from the parents or legal representatives and from the patients older than 12 years, within 24 h after start of study procedures.

Findings will be submitted to peer-reviewed journals for publication, and to local and international conferences. As we have multiple secondary outcomes, we expect to submit multiple publications to peer-reviewed journals. Findings will be communicated to the public through media coverage and personal website(s).

## Discussion

Previous studies have indicated that beta-lactam antibiotics might not achieve the PDT in critically ill children. The EXPAT Kids study aims to identify the percentage of patients which achieve target attainment and potential risk factors influencing this outcome. Our study has already been conducted in the adult population, in which male patients with higher creatinine clearance, higher serum albumin, higher white blood cell count, higher length, and lower urea and those who received concomitant antibiotics were more likely not to achieve the PDT (Abdulla et al., 2020a).

Several studies have used scavenged samples and concluded that the strategy was suitable for the establishment of popPK models (Leroux et al., 2016; Dong et al., 2018; Hahn et al., 2019; Tang et al., 2019; Shi et al., 2020; Wang et al., 2020; Wu et al., 2020; Zhao et al., 2020). The strategy is feasible when considering the generalizability in context of sampling density, quality, and stability (Leroux et al., 2015). This study will use scavenged sampling to validate the use of residual material from heparinized astrup syringes for antibiotic quantification. If validated, residual material, even in small volumes, may enrich data collection and reduce workload for medical staff.

Based on study results, a randomized controlled trial in which therapeutic drug monitoring with model-based guidelines might be the next step to assure improved clinical outcomes due to beta-lactam pharmacodynamics targets are attained. A similar study, which is finishing the inclusion phase, is currently conducted at the Erasmus Medical Center, namely the DOLPHIN Study (Abdulla et al., 2020b).

More information about the PK parameters of beta-lactam antibiotics in the critical ill pediatric population is warranted. The EXPAT Kids study’s findings may lead to new insights to improve clinical outcomes and dosing regimens of beta-lactams.
